# Predictors of Pneumococcal Co-infection for Patients with Pandemic (H1N1) 2009

**DOI:** 10.3201/eid1708.101673

**Published:** 2011-08

**Authors:** Mar Masiá, Sergio Padilla, Pedro Antequera, José Manuel Ramos, Montserrat Ruiz, Félix Gutiérrez

**Affiliations:** Author affiliations: Hospital Universitario de Elche, Elche, Spain (M. Masiá, S. Padilla, J.M. Ramos, M. Ruiz, F. Gutiérrez); University Miguel Hernández, Elche (F. Gutiérrez);; Hospital Universitario de San Juan, Alicante, Spain (P. Antequera)

**Keywords:** respiratory infections, viruses, bacteria, influenza, pneumococcal, pandemic, Streptococcus pneumoniae, influenza A, H1N1, dispatch

## Abstract

We conducted a systematic investigation of pneumococcal co-infection in patients with a diagnosis of pandemic (H1N1) 2009 and any risk factor for complications or with severity criteria. We found 14% prevalence, with one third of patients having nonpneumonic infections. A severity assessment score >1 and high C-reactive protein levels were predictors of pneumococcal co-infection.

Influenza virus and *Streptococcus pneumoniae* are 2 of the most frequently implicated pathogens in respiratory tract infections in humans. Although an interaction between these microorganisms has been suggested (*1**–**8*), few data are available addressing the prevalence, clinical spectrum, or predictive factors of pneumococcal co-infection for patients with influenza.

We investigated the prevalence and clinical characteristics of pneumococcal infection in patients infected with pandemic (H1N1) 2009 virus. We compared those patients with patients in whom only influenza or pneumococcal infection was diagnosed during the pandemic to identify potential predictors of co-infection.

## The Study

This prospective study was conducted in Spain from July 2009 through March 2010 during the outbreak of pandemic (H1N1) 2009. All adult (>18 years of age) patients with an influenza-like illness who sought medical attention and had >1 risk factor for contracting influenza-related complications, according to Centers for Disease Control and Prevention recommendations (www.cdc.gov/flu/about/disease/high_risk.htm) or any other severity criteria, were sent to an outpatient infectious diseases clinic and were included in a protocol described below.

The protocol included the collection of oropharyngeal and nasopharyngeal swab samples, tested as previously described (*9*). All patients were asked for a urine sample for pneumococcal antigen detection. A sputum sample was obtained if spontaneously expectorated, and 2 blood cultures were collected when low systolic blood pressure, hypo- or hyperthermia, signs of sepsis, or a score >2 obtained by using the British Thoracic Society’s CURB-65 assessment tool (*10*) were present. Patients had chest radiographs taken, C-reactive protein (CRP) measured, and the severity of illness calculated by using the CURB-65 score.

The CDC real-time reverse transcription PCR (rRT-PCR) protocol for detection and characterization of the pandemic (H1N1) 2009 virus was used for the diagnosis of influenza cases. The BinaxNOW *S. pneumoniae* urinary antigen test (Inverness Medical Diagnostics, Princeton, NJ, USA) was performed and read as previously described (*11*). Criteria for diagnosis of *S. pneumoniae* infection required isolation of the microorganism from blood, isolation of the predominant organism from a qualified sputum sample (*11*), or a positive urinary antigen test result.

A total of 418 patients with an influenza-like illness were evaluated, of whom 179 were confirmed as having cases of pandemic (H1N1) 2009 virus by rRT-PCR. Of these, 99 (55.3%) patients provided a urinary sample for pneumococcal antigen detection, 37 (20.7%) a sputum sample, and 48 (26.8%) blood cultures. There were no significant differences in demographic or clinical data among patients with or without a valid urinary sample for diagnostic testing, with the exception of pneumonia, which was more frequent among patients with an available sample (32.3% vs. 3.8%, p<0.001). Of 239 patients with rRT-PCR negative for influenza, pneumococcal infection was investigated in 171, of whom 43 (25.1%) had pneumococcal disease.

Of 100 patients who had influenza, a test available for pneumococcal detection, and no other bacterial pathogens identified, 14 had pneumococcal infection diagnosed (14%, 95% confidence interval 7.2–20.8; p = 0.03, compared with patients without influenza). Of these, 13 had a positive urinary antigen test result; 8 (57.1%) of the 14 had infection diagnosed only on the basis of this result. Of the remaining 6 patients, *S. pneumoniae* was isolated from blood in 2 and in sputum from 4. Demographic characteristics did not differ between pneumococcal–co-infected and non–co-infected patients (Table). Compared with patients with influenza infection only, those with pneumococcal co-infection more frequently had pneumonia (p<0.001), were more frequently admitted to hospital (p<0.001) and to the intensive care unit (p = 0.034), had lower O_2_ saturation (p = 0.006) and higher axillary temperature (p = 0.009), and more frequently had the following CURB-65 score criteria: confusion (p<0.001), respiratory rate >30 breaths/min (p = 0.009), and systolic blood pressure <90 mm Hg (p = 0.03) (Table). CURB-65 score was >1 for 35.7% of patients with pneumococcal co-infection but only 3.5% of those with influenza infection only (p<0.001). Levels of CRP were significantly higher in patients with influenza plus pneumococcal disease (190.7 mg/L vs. 26.6 mg/L; p <0.001).

**Table:** Demographic and clinical data of patients with pandemic (H1N1) 2009 virus, by pneumococcal co-infection status, Spain, July 2009–March 2010*

Variable	Influenza plus pneumococcal disease, n = 14	Influenza without pneumococcal disease, n = 86	p value†	Influenza plus pneumococcal pneumonia, n = 9	Influenza plus nonpneumococcal pneumonia, n = 15	p value†
Female sex	8 (57.1)	41 (47.7)	0.511	6 (66.7)	7 (46.7)	0.341
Age, y	42.1 (35.7–57.0)	39.2 (27.3–52.1)	0.371	39.7 (31.1–63.1)	43.6 (31.7–51.3)	0.788
Concurrent conditions‡	10 (71.4)	71 (82.6)	0.325	7 (77.8)	11 (73.3)	0.808
Pregnancy	1 (7.1)	1 (1.1)	0.188	1 (11.1)	0	0.261
Decompensated concurrent condition	3 (21.4)	15 (17.4)	0.628	2 (22.2)	5 (33.3)	0.604
HIV infection	0 (0)	8 (9.3)	0.303	0	2 (13.3)	0.280
Smoker	3 (21.4)	21 (24.4)	0.908	2 (22.2)	3 (20.0)	0.782
Alcoholism	2 (14.2)	6 (6.9)	0.293	1 (11.1)	1 (6.66)	0.674
Hospital admission	12 (85.7)	21 (24.4)	0.000	9 (100)	11 (73.3)	0.090
ICU admission	2 (14.3)	2 (2.3)	0.034	2 (22.2)	1 (6.7)	0.265
Pneumonia	9 (64.3)	15 (17.4)	0.000	9 (100)	15 (100)	NA
Pneumococcal bacteremia	2 (14.2)	NA	NA	1 (11.1)	0	0.303
Oliguria/anuria	0 (0)	1 (1.1)	0.694	0	0	NA
O_2_ saturation <94%	3 (21.4)	12 (13.9)	0.403	2 (22.2)	7 (46.7)	0.311
Axillary temperature, °C	38.1 (37.5–38.9)	36.8 (36.4–37.8)	0.009	37.7 (37.5–38.8)	36.5 (35.9–37.8)	0.679
O_2_ saturation, %	95 (85.2–95.5)	96.3 (94–98)	0.006	93 (72–95)	93 (78–110)	0.308
CURB-65 score	0 (0–2)	0 (0–1)	0.201	2 (0–2.5)	0 (0–1)	0.156
Confusion	4 (28.6)	1 (1.2)	0.000	4 (44.4)	0	0.005
BUN level >20 mg/dL	2 (14.3)	4 (4.7)	0.628	2 (22.2)	4 (26.7)	0.808
Respiratory rate >30 breaths/min	3 (21.4)	3 (3.5)	0.009	3 (33.3)	1 (6.7)	0.090
BP <90/60 mm Hg	2 (14.3)	2 (2.3)	0.034	2 (22.2)	2 (13.3)	0.572
Age >65 y	2 (14.3)	4 (4.7)	0.159	2 (22.2)	1 (6.7)	0.265
CURB-65 score >1	9 (64.3)	62 (72.1)	0.550	5 (55.5)	7 (46.7)	0.673
CURB-65 score >2	5 (35.7)	3 (3.5)	0.000	5 (55.5)	1 (6.7)	0.007
C-reactive protein, mg/L	190.7 (74.0–190.7)	26.6 (11.80–79.35)	0.000	255 (134–320)	89 (60–162)	0.008
Procalcitonin, ng/mL§	1.25 (0.12–26.00), n = 6	0.5 (0.1–0.5), n = 19	0.198	13.5 (0.59–27.70), n = 4	0.5 (0.08–6.03), n = 3	0.289
Death	0	0	NA	0	0	NA

*All values are number (%) for categorical variables and median (interquartile range) for continuous variables. NA, not applicable; ICU, intensive care unit; CURB-65, confusion of new onset, urea greater than 7 mmol/L (blood urea nitrogen [BUN] >19), respiratory rate >30 breaths/min, systolic blood pressure (BP) <90 mm Hg or diastolic blood pressure <60 mm Hg, age >65 (*10*). †The χ^2^ or Fisher exact test was used for categorical variables and the Mann-Whitney test for continuous variables. ‡Asthma, chronic lung disease, heart disease, neurologic and neurodevelopmental conditions, blood disorders, endocrine disorders (such as diabetes mellitus), kidney disorders, liver disorders, metabolic disorders, weakened immune system, people <19 y of age who are receiving long-term aspirin therapy, people who are morbidly obese (body mass index >40). §Measured in patients with signs of sepsis; n values as shown.

When only influenza cases with pneumonia were analyzed and those with pneumococcal co-infection (n = 9) were compared with patients in whom only influenza was identified (n = 15), patients with pneumococcal co-infection more frequently had confusion (p = 0.005), a CURB-65 score >1 (p = 0.007), higher CRP levels (255 mg/L vs. 89 mg/L, p = 0.008), and a statistical trend to tachypnea >30 (p = 0.09) and to higher hospital admission (p = 0.09) (Table).

Pneumococcal infection characteristics were also compared between patients with and without (n = 43) pandemic (H1N1) 2009 infection included in the study. Confusion according to CURB-65 criteria was more frequent among patients with both infections (p = 0.003), while other clinical data did not differ between groups.

## Conclusions

We found that the prevalence of concurrent pneumococcal infection was 14% in patients who had pandemic (H1N1) 2009 virus infection with any risk factor for influenza-related complications or who met severity criteria. Of note, infection more than half these patients would not have been diagnosed if a pneumococcal urinary antigen test had not been performed. We evaluated the frequency of pneumococcal disease in patients with the pandemic (H1N1) 2009 virus through a systematic investigation by using validated diagnostic methods. Although a recent study identified a high frequency of *S. pneumoniae* and *Haemophilus influenzae* in nasopharyngeal swabs from patients with influenza A (H1N1) tested by using molecular techniques (*12*), their clinical significance and positive predictive value remain undetermined.

We characterized the clinical spectrum of pneumococcal infection accompanying influenza pandemic. Moreover, the systematic calculation of the CURB-65 score provided us a simple, objective, and useful tool for categorization of severity and comparison between patients. Although the prevalence of pneumococcal infection in patients with influenza might have been overestimated by more frequent urinary sampling among those with pneumonia, we found that more than one third of the patients had nonpneumonic pneumococcal infections. Compared with patients with influenza only, pneumococcal co-infected patients showed a higher severity of disease as defined by a higher frequency of CURB-65 criteria, lower O_2_ saturation, and more frequent admission to the intensive care unit. Furthermore, a CURB-65 score >1 was found to be a predictive factor of pneumococcal co-infection. Additionally, levels of CRP were also much higher in patients with pneumococcal infection. Although many co-infected patients also had pneumonia, a fact which might have explained the above-mentioned findings, the same predictive factors distinguished between influenza pneumonia and influenza plus pneumococcal pneumonia.

Pneumococcal infection was more prevalent among patients with a negative test result for influenza. This prevalence could be explained because, presumably, many of the noninfluenza patients met the influenza-like illness definition due to bacterial infection. However, it is unknown whether some of the influenza-attributable pneumococcal infection might have been underestimated, because *S. pneumoniae* might follow influenza after a lag period, and a delay in the request for medical attention might diminish the sensitivity of diagnostic tests for influenza. Nonetheless, data from treated patients have shown a median duration of viral shedding of 5 to 9 days and slower viral clearance with delayed antiviral drug administration (*13*).

In summary, the prevalence of pneumococcal co-infection during the influenza A (H1N1) 2009 pandemic was noteworthy, and it was associated with a higher severity of disease. In one third of the cases the clinical signs and symptoms did not indicate pneumonia, and more than one half could only be diagnosed with the urinary antigen test. A CURB-65 score >1 and CRP levels proved to be useful tools to identify patients at higher risk for pneumococcal co-infection for whom physicians should adopt additional diagnostic and therapeutic measures.
